# Influence of Biological, Social and Psychological Factors on Abnormal Eating Attitudes among Female University Students in Brazil

**Published:** 2010-04

**Authors:** Larissa da Cunha Feio Costa, Francisco de Assis Guedes de Vasconcelos, Karen Glazer Peres

**Affiliations:** ^1^ Programa de Pós-Graduação em Nutrição, Centro de Ciências da Saúde; ^2^ Grupo de Estudos de Odontologia em Saúde Coletiva, Programa de Pós-graduação em Saúde Pública, Universidade Federal de Santa Catarina, Florianópolis–Santa Catarina, Brazil

**Keywords:** Anorexia nervosa, Attitudes, Body-image, Calorie intake, Cross-sectional studies, Eating disorders, Exploratory studies, Nutritional status, Brazil

## Abstract

The objective of the study was to estimate abnormal eating attitudes influenced by associated factors among female students of the Universidade Federal de Santa Catarina, Florianópolis, southern Brazil. Abnormal eating attitudes were investigated using the eating attitudes test (EAT-26), according to the presence (EAT+) and absence (EAT-) of symptoms in a sample of 220 students. The body-image was assessed by the body-shape questionnaire (BSQ-34). Body mass index, body-fat percentage, waist-circumference, food intake (24-hour food recall), and socioeconomic characteristics (monthly household income, monthly per-capita income, and parental schooling) were also investigated. Statistical associations were tested by multivariate Poisson regression analysis. The prevalence of EAT+ and dissatisfaction with the body-image were 8.3% [confidence interval (CI) 95% 4.6–12.0] and 20.0% (CI 95% 14.7–25.3) respectively. Dissatisfaction with the body-image maintained its independent association with abnormal eating attitudes, indicating symptoms of anorexia nervosa. The results of this work highlight the importance of the planning of nutrition-education programmes in universities, aiming at assisting in the choices of food that comprise a healthful diet in a period of life of so many changes and decisions.

## INTRODUCTION

Eating disorders have received progressively more attention from health professionals since these can cause significant morbidity and mortality. Eating disorders have grown over recent decades, affecting distinct countries, cultures, and socioeconomic groups, to the extent of being recognized as one of the main public-health problems today (1, 2).

Anorexia nervosa as an eating disorder was first described in the 19th century, and being the first to be adequately classified and for which the operational criteria were recognized in the 1970s. It is categorized by, among other symptoms, rapid and deliberate loss of weight brought on by extremely strict diets, together with an unrestrained desire for thinness, gross distortion of the body-image, and changes in the menstrual cycle. It starts typically from mid- to late-adolescence, and around 95% of cases occur in women (3, 4).

The currently-accepted aetiological model is multi-factorial, with contributions coming from psychological, biological, familial and sociocultural factors, all of which interact to determine the mani-festation of anorexia nervosa(1–3).

Anorexia nervosa is an illness that leads to stunted growth, with excessive loss of weight and great physical and psychological impact. Numerous complications occur in affected individuals, including dehydration, cardiac impairment, disturbances of gastrointestinal motility, renal problems, infertility, hypometabolism, dental problems, anaemia, and osteoporosis. Patients who are in pre-puberty may exhibit a delay in sexual maturation, in physical development, and in growth and may not reach their expected height (5, 6).

The factors triggering the eating disorders may be a significant event, such as loss of a loved one, separation, changes in family dynamics, organic disease, body-image disturbances, depression, anxiety, or even childhood trauma, such as sexual abuse. However, the way in which these factors act to cause the disorder remains to be clarified (7, 8).

Studies among university students have shown that they may compromise their nutritional status as a result of inadequate nutrition caused by dietary fads and skipping meals, among other factors (9). According to Cunha and Carrilho, the first year in higher education is a phase of challenge for students since it represents a period of development and academic adjustment, which demands adaptation and integration into the new environment (10). Vieira *et al*. found that 15% of university students began to exhibit health problems after entering university and attributed such disturbances to emotional causes, suggesting that the changes inherent in beginning university life could have affected the emotional balance and health in this group of individuals (11).

Despite the growing importance of the theme, studies among the university population are barely found, especially in developing countries. Epidemio-logical studies are important to define preventive health policies. The objectives of this study were to estimate the abnormal eating attitudes associated with biological, social and psychological factors and to describe the patterns of food intake by first-year students at the Universidade Federal de Santa Catarina (UFSC), Brazil, in 2006.

## MATERIALS AND METHODS

A cross-sectional, exploratory study was carried out, involving female adolescents and young adults (n=935) in their first semester in 55 undergraduate courses at the UFSC. The UFSC, located in Florianópolis, southern Brazil, is considered one of the largest and most important federal universities in Brazil.

The following parameters were adopted to calculate the minimum sample size: 95% confidence level, a sample error of 2.85 percentage points, and a prevalence of symptoms of anorexia nervosa of 5.5% (12), which resulted in a sample size of 212 students. The calculations included an addition of 20% to compensate non-responses. The minimum required sample size was 254 students. The subjects were systematically selected from a complete list, including all female undergraduate students in an alphabetical order. Being pregnant or physically handicapped was considered an exclusion criterion. However, neither condition was observed in our study.

Data were collected during 5 June–8 December 2006.

### Independent variables

The following variables were investigated: age; maternal and paternal schooling in terms of complete years of study; number of persons in the family; and monthly income of students and their domestic living arrangements.

The age of the subjects in years was calculated by subtracting the date of birth from the date of data collection, and the students were allocated in two age-groups (16–19 years and ≥20 years).

The variables—maternal and paternal schooling—were categorized as follows: 1–4 years, 5–8 years, 9–12 years, and more than 12 completed years of study.

The monthly per-capita income was obtained by dividing the self-reported monthly household income by the number of inhabitants in the family. The monthly household income was converted to minimum wages and then categorized as: less than 1, 1.1–3, 3.1–6, and ≥6.1. One minimum wage in Brazil corresponds to US$ 201.14, based on the exchange rate as in December 2007 (US$ 1=Reais 1.74).

Domestic arrangements were categorized as: living alone and with parents or colleagues. The practice of physical activity was classified as: no exercise undertaken, exercise undertaken less than three times a week, and exercising three or more times per week.

### Body-image

The presence of worries concerning the body-image was investigated by means of the body-shape questionnaire (BSQ-34)—in the version translated into Portuguese by Cordás and Castilho (13). Students who scored from 0 to 80 points were classified as having no dissatisfaction with the body-image; 81 to 110 points slight worry; 111 to 140 moderate worry, and 141 to 204 points being classified as severe worry (14). The variable was later dichotomized as BSQ negative (BSQ-), for those who scored from 0 to 110 points, and BSQ positive (BSQ+) for those with a score above 111 points, for statistical analysis.

The BSQ is a self-applied test, which in its first validation study proved to be satisfactory for the assessment of concerns over the body-image, reduced self-worth due to physical appearance, and the sensation of being fat and includes as a parameter the eating disorder inventory (EDI) subscale of dissatisfaction over body-image with a total score of EAT-26 (14).

Rosen *et al*. found a significant reliability coefficient of 0.88 for all the 34 items of psychometric characteristics on the BSQ-34 test (15). Similar results were observed in Brazil, after validation of the instrument in Portuguese (16).

### Food intake

Food intake was examined by calculating the energy intake in kilocalories and that of macronutrients in grammes (proteins, lipids, and carbohydrates), collected through the 24-hour food recall. These data were recorded using the Nutwin software (version 2.5), developed by the Health Informatics Centre of the Universidade Federal de São Paulo, Brazil. The daily intake of each nutrient was analyzed, according to the recommendations of the dietary reference intakes for energy, carbohydrates, fibre, fat, protein, and acids (macronutrients) (17).

### Anthropometry

The following measurements were taken: weight, height, waist-circumference, and bicipital, tricipital, subscapular and suprailiac skin-folds. These data were collected according to Lohman *et al*. (18). During data collection, the students were instructed to remove shoes, belts, heavy coats, jackets, and thick shirts.

The weight, height, and waist-circumference were measured only once while the tricipital, bicipital, subscapular and suprailiac skin-folds were obtained at three distinct time points, measured by one of the principal researchers who had been previously trained by a physical education professional with the aim of avoiding variability among the measurements.

The weight was determined using an electronic balance with a capacity of 180 kg and a precision of 100 g. The height was obtained using a portable anthropometer with a bilateral scale from 35 to 213 cm and a resolution of 0.1 cm. The waist-circumference was measured using an extendable metric tape with a precision of 1 mm.

The skin-folds were measured with a Lange scientific adipometer with a precision of 1 mm.

The weight and height of the subjects were employed to calculate the body mass index (BMI), diagnosed from the criterion recommended by the World Health Organization (WHO) (19), which regards as underweight values for BMI <18 kg/m^2^, with a eutrophic BMI between 18.5 and 25 kg/m^2^, overweight BMI between 25 and 30 kg/m^2^ and obese BMI ≥30.0 kg/m^2^.

The waist-circumference analysis followed the criteria recommended by the WHO (19), which consider a waist-circumference of ≥80 cm as a risk factor for abdominal obesity.

Measurements of bicipital, tricipital, subscapular and suprailiac skin-fold thickness were used for estimating the percentage of body-fat according to the formula of Durnin and Womersley (20). The classification followed the criteria proposed by Lohman (21), which consider eutrophic as a percentage of body-fat between 9 and 31, subnutrition equal to or less than 8, and obesity as equal to or greater than 32.

### Dependent variable: abnormal eating attitudes

Abnormal eating attitudes were identified through the eating attitudes test (EAT-6) (22), translated into Portuguese by Nunes *et al*. (23). Students who scored 20 points or more on the EAT-26 were classified as individuals who exhibited dietary habits, suggestive of abnormality, i.e. symptomatic of anorexia nervosa (EAT+). Those who scored less than 20 were classified as symptom-free (EAT-).

The EAT-26 is a self-report instrument developed by Garner and Garfinkel to assess and identify abnormal eating standards (22). It is a psychometric test to measure symptoms of anorexia nervosa easily and quickly, thereby favouring early diagnosis and treatment and preventing the evolution of the disease (24).

In Brazil, the first translation was performed by Nunes *et al*., and this was tested in adolescents, aged 12–15 years, from public schools, with the result being considered to be adequate (23).

Magalhães and Mendonça performed a reliability test for the EAT-26, with a sample of 60 first-year students at a public university in Rio de Janeiro (25). The value of Kappa obtained for this instrument was 0.81.

A pilot study was performed to test the instruments and identify the possible difficulties during the data-collection stage. Students from the nutrition course, who were not in their first semester, were assessed, enabling the optimization of anthropometric measurement techniques.

Socioeconomic and dietary variables and information regarding dissatisfaction with the body-image and abnormal eating attitudes were collected using a questionnaire, filled up by the subjects receiving guidance on its correct completion.

The initial contact in performing the study was made through the course coordinators. Later, members of teaching staff were approached, and they gave a few minutes of their classes for an explanation of the study, followed by the presentation of objectives, subject-selection method, and research activities. Contact with the students was attempted on three occasions, and if there was no response, they were regarded as losses.

To avoid any possible bias in sampling, it was decided not to substitute the students who declined to participate or who did not attend for data collection.

### Statistical analysis

Statistical calculations were performed using the Stata software (version 9.0). Descriptive statistics were calculated giving the distributions of relative and absolute frequencies for the variables of interest in the study and means and respective standard deviations for the quantitative variables. Pearson's chi-square test was applied to test the association between the independent variables and the outcome. To analyze the potential risk factors for the presence of abnormal eating attitudes, Poisson's multiple regression analysis was carried out, and the prevalence ratios with 95% confidence interval were obtained. A value of p<0.25 in bivariate analysis was used for selecting variables for insertion in the multiple model, to control for possible confounding variables. Variables with p<0.05 were retained in the final model.

### Ethics

The Committee for Ethics in Research in Humans at the UFSC approved the research protocol.

## RESULTS

The response rate achieved in the study was 86.6%. The prevalence of abnormal eating attitudes among the first-year students was 8.3% (CI 95% 4.6–12.0).

Table 1 shows the general characteristics of the sampled subjects. The table abo shows that the mean age of the study subjects was 20.2 years (standard deviation 2.75).

**Table 1.** Distribution of values for measures of central tendency and dispersion for demographic, socioeconomic, and anthropometric variables and food intake among first-year university students in the first semester of 2006, UFSC, Florianópolis (Santa Catarina)

Variable	No.	Mean	Standard deviation
Age (years)	220	20.2	2.75
Maternal schooling (years of study)	219	12.3	4.07
Paternal schooling (years of study)	217	12.6	4.52
No. of family members	219	4.05	1.20
Monthly household income (Reais*)	190	4,469.90	3,807.12
Monthly percapita income (Reais*)	190	1,128.70	889.46
Weight (kg)	220	57.2	9.96
Height (cm)	220	163.7	6.52
Body mass index (kg/m^2^)	220	21.3	3.38
Waist-circumference (cm)	220	69.0	7.24
Breastfeeding (%)	220	28.0	4.10
Energy intake (kcal)	219	1,780.86	747.96
Protein intake (g)	219	72.2	36.65
Carbohydrate intake (g)	219	239.0	113.08
Lipid intake (g)	219	62.1	35.76

*US$ 1=Reais 1.74 as in December 2006;

FSC=Universidade Federal de Santa Catarina

The mean number of completed years of maternal and paternal schooling was 12. However, when these data were categorized in years of schooling, it was revealed that 82.3% of mothers (n=180) presented more than eight years of schooling while 78.3% of fathers (n=170) had more than eight years of schooling.

The mean monthly per-capita income was Reais 1,128 (US$ 648.27). The mean energy intake in the subjects was 1,780.86 kcal, with a standard deviation of 747.96.

Table 2 presents the distribution of the independent variables, according to abnormal eating attitudes. The table shows that the group of first-year students dissatisfied with their body-image exhibited a significantly greater prevalence of abnormal eating attitudes (34.2%) when compared with the group of students satisfied with their body-image (2.3%).

**Table 2.** Prevalence of abnormal eating attitudes and association with behavioural variables, classification of nutritional status, and dissatisfaction with body-image among first-year university students in the first semester of 2006, UFSC, Florianópolis (Santa Catarina)

Variable	Sample distribution (%)	Anorexia nervosa (%)	95% CI
Body-image			
Body-shape questionnaire-	80.0	2.3	0.0–4.5
Body-shape questionnaire+	20.0	34.2	19.0–49.3
Monthly household income (MS)*			
≥6.1	72.1	5.8	1.9–9.8
3.1–6.0	17.9	9.4	0.0–20.1
1.1–3.0	10.0	5.3	0.0–16.3
Living with			
Relatives	69.6	8.7	4.1–13.2
Colleagues	20.9	6.5	0.0–13.9
Alone	9.5	9.5	0.0–23.2
Age (years)			
Up to 19.9	57.7	9.5	4.3–14.7
20.0 or above	42.3	6.6	1.3–11.8
Physical activity (days per week)			
≥3	27.3	8.3	1.1–15.1
<3	16.8	13.9	2.0–25.8
Not undertaken	55.9	6.6	2.1–11.1
Nutritional status (BMI)			
Underweight (<18.5 kg/m^2^)	15.9	2.9	0.0–8.7
Eutrophic (between 18.5 and 25 kg/m^2^)	72.3	10.3	5.4–15.1
Overweight and obese (≥25 kg/m^2^)	11.8	3.9	0.0–11.8
Energy intake (kcal)			
≥2,200	85.8	5.4	2.1–8.7
<2,200	14.2	22.6	7.0–38.2

*MS=Minimum salary (1 minimum salary is equivalent to Reais 350.00 in May 2006, approximately US$ 201.14);

BMI=Body mass index;

CL=Confidence interval;

UFSC=Universidade Federal de Santa Catarina

The prevalence of abnormal eating attitudes was greater among those students whose monthly household income was found to be between 3.1 and 6.0 minimum salaries (1 minimum salary is equivalent to Reais 350.00 in May 2006, approximately US$ 201.14) but this difference was not significant.

The highest prevalence of abnormal eating attitudes was found among students who lived alone (9.5%) when compared with the other categories, although there was no association among these variables (p=0.907).

Furthermore, Table 2 shows that there was no significant relationship among the students positively identified with abnormal eating attitudes in terms of age, or whether the subject did or did not take part in physical activity.

The students considered to be eutrophic exhibited a greater prevalence of abnormal eating attitudes (10.3%) when compared with those who were underweight (2.9%).

The Poisson's multiple regression analysis demonstrated that the prevalence of abnormal eating attitudes was significantly higher among students with a low energy intake (<2,200 kcal); however, this association lost its statistical significance in the adjusted analysis (Table 3). Dissatisfaction with the body-image was the only variable that presented an independent association with the presence of other variables analyzed. The students dissatisfied with their body-image exhibited prevalence of abnormal eating attitudes 13.5 times greater than those satisfied with their body-image.

**Table 3.** Poisson's regression analysis of abnormal eating attitudes and independent variables. First-year university students in the first semester of 2006, UFSC, Florianópolis (Santa Catarina)

Variable	Raw PR (95% CI)	p value	Raw PR (95% CI)	p value
Body-image		<0.001		<0.001
Body-shape questionnaire-	1.0		1.0	
Body-shape questionnaire+	15.0 (5.2–43.4)		13.5 (3.5–51.7)	
Monthly household income (MS)†		0.796		
≥6.1	1.0		e*	
3.1–6.0	1.6 (0.4–5.7)			
1.1–3.0	0.9 (0.1–6.8)			
Living with		0.907		
Relatives	1.0		e*	
Colleagues	0.8 (0.2–2.5)			
Alone	1.1 (0.3–4.5)			
Age (years)		0.445	e*	
Up to 19.9	1.0			
20.0 or over	0.7 (0.3–1.8)			
Physical activity (days per week)		0.535	e*	
Undertaken ≥3	1.0			
Undertaken <3	1.7 (0.5–5.4)			
Not undertaken	0.8 (0.3–2.3)			
Nutritional status		0.202		0.241
Underweight (BMI <18.5 kg/m^2^)	0.3 (0.1–2.0)		0.8 (0.1–6.9)	
Eutrophic (BMI ≥18.5 and <25 kg/m^2^)	1.0		1.0	
Overweight and obese (BMI ≥25 kg/m^2^)	0.4 (0.1–2.7)		0.3 (0.1–4.7)	
Energy intake (kcal)		0.002		0.220
≥2,200	1.0		1.0	
<2,200	4.2 (1.7–10.2)		1.9 (0.7–5.0)	

e*=Excluded from bivariate analysis;

†MS=Minimum salary (1 minimum salary equivalent to Reais 350 in May 2006, approximately US$ 201.14);

BMI=Body mass index;

CL=Confidence interval;

PR=Poisson regression;

UFSC=Universidade Federal de Santa Catarina

## DISCUSSION

The results of the present study are based on a probabilistic representative sample of students entering the university in the first semester of 2006 in 55 undergraduate courses at the UFSC. The method adopted for the calculation and selection of the sample in the study reinforces the internal validity of the results. The sample investigated did not also differ significantly from the original population.

The prevalence of abnormal eating attitudes observed in the study (8.3%) is within the range reported in other studies of the university population carried out in Brazil, which employed the EAT-26 to identify abnormal eating attitudes (12, 27). The worldwide prevalence of anorexia nervosa varies with the sample and the assessment methods. According to study data provided by the American Psychiatric Association (APA), the prevalence of anorexia was found to vary from 0.5% to 3.7% (5). In Brazil, studies have focused on the identification of abnormal eating attitudes in populations. A search for studies that investigated abnormal eating attitudes in Brazilian samples identified nine examples carried out during 2001–2006, in which the prevalence varied markedly from 4.7% to 21.1% (6, 12, 27–30). The observed inconsistencies in the results of studies may be partially explained by the differences in age range, cultural background, and different levels of exposure of subjects to well-known risk factors. In the municipality of Florianópolis (SC), the prevalence of abnormal eating attitudes was found to be 15.6% among female students aged 10–19 years (30).

It is important to emphasize that the students investigated had not been diagnosed with anorexia nervosa. The study merely estimated the presence of symptoms of this problem according to the EAT-26, which identifies abnormal dietary standards. The EAT is a screening measure to help determine if individuals may present an eating disorder that requires professional attention.

This probably explains why most (88.9%) subjects with abnormal eating attitudes were classified as eutrophic. Moreover, the prevalence of abnormal eating attitudes was the highest among students with an energy intake below the average daily energy requirement for women in the age-group (31). This corroborates the findings of studies among university students which showed that they may compromise their nutritional status through inadequate nutrition characterized by dietary fads and skipped meals, among other factors (9).

Dissatisfaction with the body-image was the only variable that showed a significant association with the presence of abnormal eating attitudes, suggesting symptoms of anorexia nervosa. In the study by Alves *et al*., dissatisfaction with the body-image was also the strongest risk factor for the presence of abnormal eating attitudes (30). Similarly, Nunes *et al*. reported that dissatisfaction with the body-image was the most important factor in the causation of eating disorders (28).

Some studies have drawn attention to an increase in the prevalence of eating disorders. Dunker and Phillippi observed that the increase in the prevalence of anorexia nervosa has occurred at the same time that a greater emphasis has been placed on female thinness as an expression of sexual attraction, in which society values attractiveness and thinness in particular, making obesity a highly-stigmatized and rejected condition (29). Furthermore, the model of beauty imposed by modern society, corresponding to a thin body, gives no consideration to aspects relating to health and differences in the physical make-up of the population. These observations help understand the reasons for dissatisfaction with body-image as felt by many women.

As a result, a distorted body-image has been recognized as one of the main factors in the causation of eating disorders. Fleitlich *et al*. noted that individuals with anorexia nervosa tend to see themselves as fat or out of proportion, despite being thin (1).

The factors that can interfere in perception the body-image include: social factors, sociocultural influences, media pressures, and the constant search for an ideal body associated with self-realization and happiness. All of these, including the distorted perception of the body-image, can interfere in the nutritional balance of an individual (32).

Contemporary Western societies are currently living under an ideal of thinness and of good physical shape. This standard imposes itself especially upon women, making physical appearance an important measure of personal worth. New and miraculous weight-loss diets proliferate while gyms offer countless exercise options and display the high technological investment in the development of exercise techniques (33).

Data found in the literature confirm that age-group (adolescents and young adults), sex (female) (3, 4) and dissatisfaction with the body-image are major factors associated with the presence of abnormal eating attitudes, the latter further corroborated by the results of this investigation. Furthermore, the university context is in itself a stressful factor that should be taken into consideration. In addition, many other psychological and emotional factors can play an important role in causing eating disorders that mimic symptoms of anorexia nervosa. There are undoubtedly a genetic predisposition, a range of environmental risk factors, and some information available with respect to the identity and relative importance of these contributions. However, virtually nothing is known about the individual causal processes involved, or about how they interact and vary across the development and maintenance of disorders (2).

According to Paxton, a trigger factor is usually present in the form of a significant event, such as loss of a loved one, separation, changes in family dynamics, organic disease, body-image disorders, depression, anxiety, and even childhood trauma, such as sexual abuse (7). However, the way in which these factors act to cause the disorder remains to be clarified.

The proportions of students according to type of undergraduate course, such as medicine (4.5%), architecture (7.3%), and librarianship (7.3%), were similar in the sample and among the female students at the UFSC as a whole (3%) (26). Therefore, these results should be extrapolated to other populations with caution. Another limitation is the cross-sectional design adopted in the study, which does not allow the identification of a causal relationship between the outcome and the independent variables.

As such, other variables need to be examined in further studies on this theme. Future, population-based research should be carried out to determine whether the results obtained in our study are also found on a wider scale.

The results of this work highlight the importance of the planning of nutritional education programmes in universities, with the aim of increasing the understanding of nutrition and assisting in the choices of food that comprise a healthy diet in a period of life of so many changes and decisions.

## ACKNOWLEDGEMENTS

The authors are grateful to the students who participated in and, thereby, enabled this work to be carried out and to Monalisa Cenci for her contribution in carrying out the investigation, assisting in the preparation of the research project, and data collection.
